# Novel Approach to Repeated Arterial Blood Sampling in Small Animal PET: Application in a Test-Retest Study with the Adenosine A1 Receptor Ligand [^11^C]MPDX

**DOI:** 10.1007/s11307-016-0954-9

**Published:** 2016-04-18

**Authors:** Jürgen W. A. Sijbesma, Xiaoyun Zhou, David Vállez García, Martin C. Houwertjes, Janine Doorduin, Chantal Kwizera, Bram Maas, Peter Meerlo, Rudi A. Dierckx, Riemer H. J. A. Slart, Philip H. Elsinga, Aren van Waarde

**Affiliations:** 1 University Medical Center Groningen, Department of Nuclear Medicine and Molecular Imaging, University of Groningen, Hanzeplein 1, 9713 GZ Groningen, The Netherlands; 2 Experimental Anesthesiology and Clinical Pharmacology Research Group, University of Groningen, Antonius Deusinglaan 1, 9713 AV Groningen, The Netherlands; 3 Groningen Institute for Evolutionary Life Sciences, University of Groningen, Nijenborgh 7, 9747AG Groningen, The Netherlands; 4 Department of Biomedical Photonic Imaging, University of Twente, P.O. Box 217, 7500AE, Enschede, The Netherlands

**Keywords:** Test-retest reproducibility, Positron emission tomography, Adenosine A1 receptor, Brain, Arterial blood sampling

## Abstract

**Purpose:**

Small animal positron emission tomography (PET) can be used to detect small changes in neuroreceptor availability. This often requires rapid arterial blood sampling. However, current catheterization procedures do not allow repeated blood sampling. We have developed a procedure which allows arterial sampling on repeated occasions in the same animal.

**Procedures:**

Eleven male Wistar rats were two times catheterized *via* a superficial branch of a femoral artery and scanned with [^11^C]MPDX and blood sampling. PET images were co-registered to a magnetic resonance imaging (MRI) template. Regional tracer distribution volumes (*V*_T_) in the brain were calculated by the Logan analysis. The procedure was repeated after 1 week.

**Results:**

Surgery was successful in 90 % of the cases, and discomfort was minor. The *V*_T_ data showed small differences between test and retest, low between subject variability, and a strong agreement between and within subjects.

**Conclusion:**

Repeated quantitative imaging with a high reproducibility is possible with this approach.

## Introduction

Small animal positron emission tomography (PET) is a well-established method to visualize neuroreceptors, protein aggregates, ion channels, enzymes, or transporter proteins in the brain and other organs, using radiolabeled compounds. In many cases, blood sampling is required to determine the dynamics of radiotracer clearance and metabolism for quantification of specific binding in the tissue of interest, especially when a reference tissue as input for a reference tissue model (RTM) is not available [1–3]. Catheterization of an artery is needed for rapid collection of arterial plasma during the scan. Plasma samples are used to measure radioactivity and to determine the ratio of intact parent tracer and radioactive metabolites in these samples to acquire an input function for the analysis. For arterial plasma collection, the femoral arteries are often used [4–6]. The arteries are easy accessible, required materials for the catheterization are cheap, and the impaired blood flow does not affect major organs like the brain or abdomen, but because of the invasiveness of this technique, rats must be euthanized after the scan [4–6]. This makes longitudinal studies with multiple scans in the same animal impossible.

For this reason, we have developed a cheap and technically easy surgical procedure which has the same benefits as the commonly used catheterization of a femoral artery but is minimally invasive and allows repeated catheterization and blood collection. Here, we describe this procedure and use the adenosine A1 receptor ligand [^11^C]MPDX to validate it. In order to assess variability and repeatability of the PET scans, we performed a test-retest study in which 11 rats were subjected to the novel surgical procedure, and two PET scans with the adenosine A1 receptor ligand and rapid arterial blood sampling were made in each animal, with an interval of 1 week. We show that repeated quantitative PET scans of neuroreceptors can be made in the same rat, even when rapid arterial blood sampling is required.

## Materials and Methods

### Animals

Male Wistar Unilever rats (8 ± 2 weeks of age) were obtained from Harlan (Boxmeer, the Netherlands). After delivery, the animals were acclimated for at least 7 days to recover from the transport and adapt to the new housing conditions. The rats were housed in Makrolon cages at a constant temperature of 21 ± 2 °C, maintained at a 12-h light/12-h dark regime, and fed standard chow *ad libitum*.

The experimental protocol was approved by the Institutional Animal Care and Use Committee of Groningen University (File No. 5841D). All experiments were performed by licensed investigators in compliance with the Law on Animal Experiments of The Netherlands.

### Surgery and Blood Collection

**Fig. 1:**
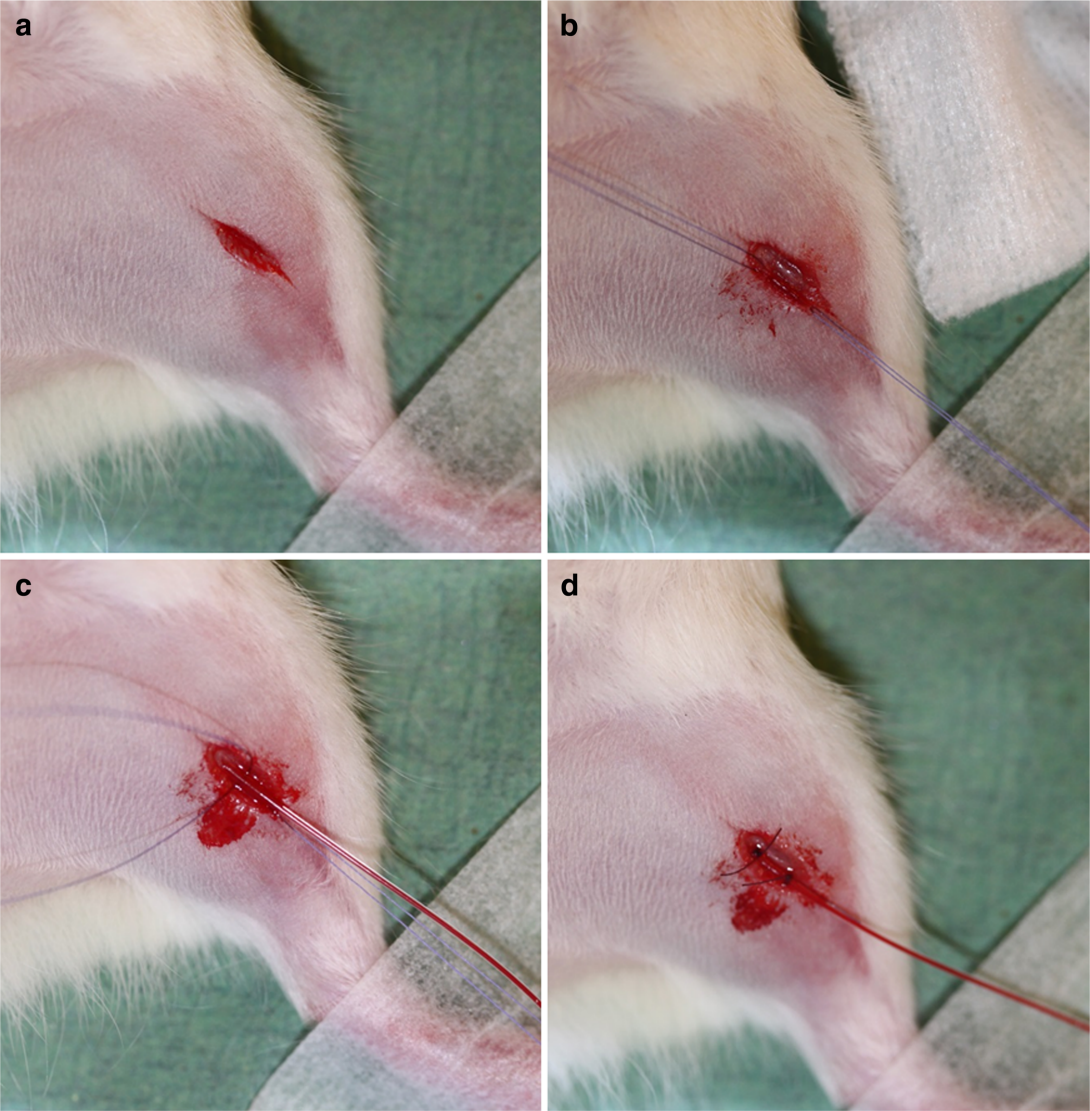
Pictures taken during the surgical procedure. **a** Incision in the left hind limb. **b** Artery is fixed with a medial and lateral suture. **c** Catheter is inserted in the artery. **d** Catheter is secured with two sutures. Artery is closed with a third suture at the lateral side.

Thirty minutes before the start of each PET scan, each rat was anesthetized using a mixture of isoflurane and medical air (5 % for induction, ≤ 2 % for maintenance; Pharmachemie BV, Haarlem, the Netherlands). The rat was placed on a heating mat connected to an electronic temperature controller with a set point of 38 °C and was positioned on its dorsal side. A 26G catheter (0.64 × 19 mm Terumo) was inserted in one of its tail veins for later injection of [^11^C]MPDX. The left hind limb of the rat was stretched out and fixed. A small (1 to 1.5 cm) incision of the skin (surgical blade no. 15, Swann-Morton REF0205) was made in the lower part of the thigh from medial to caudal-lateral side, at the height of the patella (Fig. 1). A small superficial artery (located where the femoral artery passes over in the saphenous artery) was exposed and fixed with a medial and a lateral suture (V991H Ethicon). After puncturing the vessel wall with a needle (29G 0.33 × 12 mm Terumo), a thin tube (polythene, 0.28 mm inner diameter, 0.61 mm outer diameter, REF800/100/100 Portex) was inserted and moved up through the blood vessel until the femoral artery was reached. The wound was covered with a small piece of wet (0.9 % NaCl solution) gauze. The tube and catheter were regularly flushed with a warm (37 °C) solution of saline and 1 % heparin.

Small arterial blood samples (0.1 to 0.15 ml) were manually collected at 0.17, 0.33, 0.5, 0.67, 0.83, 1, 1.5, 2, 3, 5, 7.5, 10, 15, 30, and 60 min after the start of the PET data acquisition. Drawn blood was replaced by an equal volume of saline. During the first minute after the start of tracer injection, this was done by a continuous infusion of tracer in saline *via* the venous cannula. During the period ranging from 1 to 60 min, heparinized saline was injected *via* the arterial cannula after the drawing of each blood sample. The amount of blood which was drawn from the animal (all samples combined) was less than 10 % of its total blood volume. Twenty-five microliters of whole blood was collected, and the remaining sample was centrifuged (5 min at 13,000×*g*) to obtain 25 μl of plasma. A calibrated gamma counter (CompuGamma CS1282, LKB-Wallac, Turku, Finland) was used to determine radioactivity in plasma and whole blood. Results are expressed as standardized uptake values (SUVs), defined as (plasma activity concentration [MBq/g] × body weight [g]/injected dose [MBq]).

After the PET scan and blood collection, the tube and catheter were removed and the artery was closed with three sutures. The wound was closed with three to five interrupted sutures (V991H Ethicon). Bupivacaine (Marcaine 0.5 %, AstraZeneca, 2.5 mg/kg, maximum volume of 0.6 ml) was injected subdermally at the edges of the wound. After this treatment, the rats were allowed to recover from anesthesia and surgery in a pre-warmed cage. Scanned rats were finally returned to their home cages and checked daily for weight loss, bleeding, infection, and disturbed movement.

Since the superficial artery in the left hind leg was closed, for the second scan, a catheter was inserted in the superficial branch of the femoral artery in the right hind limb, or in the main femoral artery in the left hind limb, as described previously [7].

### Radiochemistry

The radioligand [^11^C]MPDX was prepared as described previously [7], by reaction of [^11^C]methyl iodide with the appropriate 1-*N*-desmethyl precursor. The decay-corrected radiochemical yield was 35 ± 5 % (based on [^11^C]methyl iodide). Average specific radioactivity was 85 ± 35 TBq/mmol for the test scan and 93 ± 43 TBq/mmol for the retest scan. Radiochemical purity was in all cases greater than 99 %.

### PET Scans and Biodistribution Study

The rat was positioned at the center of the ring system of a microPET Focus 220 camera (Siemens Medical Solutions, USA), on its dorsal side with the brain in the field of view. Body temperature was kept close to normal using a heating mat, an electronic temperature controller, and a rectal probe. Blood oxygen levels and heart rate were continuously monitored with a pulse oximeter (Nonin PulseSense). The fraction of isoflurane in the inhaled gas mixture and the gas flow were adjusted when blood oxygen levels and heart rate decreased. A transmission scan (duration 515 s) was made with a Co-57 point source to correct the subsequently acquired emission data for attenuation and scatter. The tracer solution (39 ± 18 MBq [^11^C]MPDX in 1 ml saline) was administered during a period of 60 s, using an infusion pump (Harvard model HA1100DU). Data acquisition by the microPET camera and the infusion pump were started simultaneously. List mode data were acquired during a period of 60 min.

Exactly 1 week after the initial scan, a second PET scan with [^11^C]MPDX (26 ± 13 MBq) was made which also included arterial blood sampling. Rats were euthanized after the scan, by removal of the heart under deep general anesthesia. Several brain areas (amygdala, caudate putamen, mesencephalic region, pons, medulla, cerebellum, olfactory bulb, cortex, hippocampus, hypothalamus, thalamus, olfactory cortex, and the rest of the brain) were dissected, and peripheral organs were excised. The collected samples were weighed, and radioactivity in these samples was measured with a gamma counter.

### Data Analysis and Statistics

The software package MicroPET Manager (Siemens) was used to reconstruct the data in a dynamic frame sequence of 6 × 10, 4 × 30, 2 × 60, 1 × 120, 1 × 180, 4 × 300, and 3 × 600 s. An Ordered Subset Expectation Maximization (OSEM2D) reconstruction algorithm with Fourier rebinning, four iterations, and 16 subsets was employed obtaining images with 128 × 128 × 95 matrix, pixel width of 0.467 mm, and a slice thickness of 0.796 mm. The program PMOD version 3.5 (PMOD Technologies Ltd, Zürich, Switzerland) was used to co-register the [^11^C]MPDX PET images with a magnetic resonance imaging (MRI) template [8, 9]. Volumes-of-interest (VOI) for relevant brain regions (amygdala, caudate putamen, mesencephalic region, pons, medulla, cerebellum, olfactory bulb, cortex, hippocampus, hypothalamus, thalamus, olfactory cortex, and the rest of the brain) were defined based on the Paxinos atlas [10]. Previously acquired [^11^C]MPDX metabolite data were used to correct plasma radioactivity data for metabolites (see [7], also for experimental details). Plasma time-activity curves (TAC), whole blood radioactivity, and metabolite data were used for Logan graphical analysis [11], fit starting at 10 min, blood volume fixed at 3.6 % [7], a 1 tissue compartment model (1TCM) fit, and a 2 tissue compartment model (2TCM) fit to calculate regional tracer distribution volumes (*V*_T_). A simplified reference tissue model (SRTM), with the olfactory bulb as reference region [12], was applied to calculate tracer binding potential (*BP*_ND_) [13–15].

To show that the reproducibility of the novel approach is not affected by the template, summed static [^11^C]MPDX PET images were used to create a tracer-specific rat brain template as described in [9]. The [^11^C]MPDX PET images were also co-registered with this PET template to define VOIs and to calculate regional *V*_T_ with Logan graphical analysis.

The reproducibility between test and retest was calculated as a relative difference (Eq. (1)) and as test-retest variability (TRV) (Eq. (2)) [16]:

1 $$ \mathrm{Relative}\ \mathrm{difference} = 100*\left(\mathrm{Retest}/\mathrm{Test}\right)\hbox{--} 100. $$

2 $$ \mathrm{T}\mathrm{R}\mathrm{V}=100*\left[\mathrm{Test}\hbox{-} \mathrm{Retest}\right]/\left[\left(\mathrm{Test}+\mathrm{Retest}\right)/2\right]. $$

Variability was expressed as coefficient of variance (COV) (Eq. (3)):

3 $$ \mathrm{C}\mathrm{O}\mathrm{V} = 100*\left(\mathrm{S}\mathrm{D}/\mathrm{Mean}\right). $$

The reliability of the measurements between and within subjects (MSWS mean square between and within subjects) was expressed as intraclass correlation coefficient (ICC) (Eq. (4)). To calculate ICC, we used the two-way mixed model with the absolute agreement type and a confidence interval of 95 %. ICC values between 0.0 and 0.2, 0.3–0.4, 0.5–0.6, 0.70–0.8, and 0.9–1.0 are, respectively, considered as slight, fair, moderate, substantial, and almost perfect agreement [17].

4 $$ \mathrm{I}\mathrm{C}\mathrm{C}=\left[\mathrm{MSBS}\hbox{-} \mathrm{MSWS}\right]/\left[\mathrm{MSBS}+\left(\mathrm{n}\hbox{-} 1\right)*\mathrm{MSWS}\right]. $$

All data are presented as mean ± SD. Differences between the test and retest scans (injected dose, specific activity, tracer purity, calculated *V*_T_) were examined using a paired *t* test using SPSS (IBM SPSS Statistics 22). *V*_T_ values calculated from PET images co-registered with a MRI or a PET template and % relative difference, TRV, and ICC observed using these different templates were also compared using a paired *t* test. *p* values <0.05 were considered statistically significant.

## Results

### Surgery

**Fig. 2:**
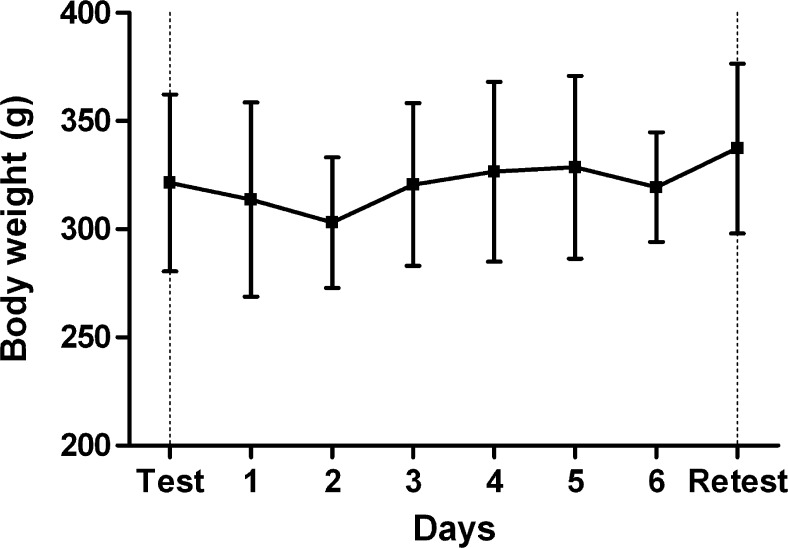
Daily body weight between the test (*day 0*) and retest scans (*day 7*). *Error bars* indicate SD.

In two animals, the superficial branch of the femoral artery was too thin for placement of a catheter, resulting in failure of the experiment. Surgery was successful in 82 % (9 out of 11) of the test scans and in 100 % (9 out of 9) of the retest procedures, thus in 90 % of all attempts. Daily inspection of the animals showed that a minor drop of body weight occurred after the initial scan, most likely due to some discomfort, but the normal rate of weight gain was resumed after 2 days (Fig. 2). No visual signs of bleeding, infection, or disturbed movement were detected, and the wound closed within 2 days, leaving only minor scar tissue. However, at the beginning of our study, two rats managed to remove their sutures on the day of the test scan, after they had woken up from anesthesia. Thus, it was necessary to anesthetize them again and to resuture their wounds. Data of these animals were excluded from the final dataset since in their case, the procedures on the test and retest days were not identical. Data from an additional rat were also excluded since that animal developed a breathing depression during the retest scan.

### Blood Sampling

**Fig. 3:**
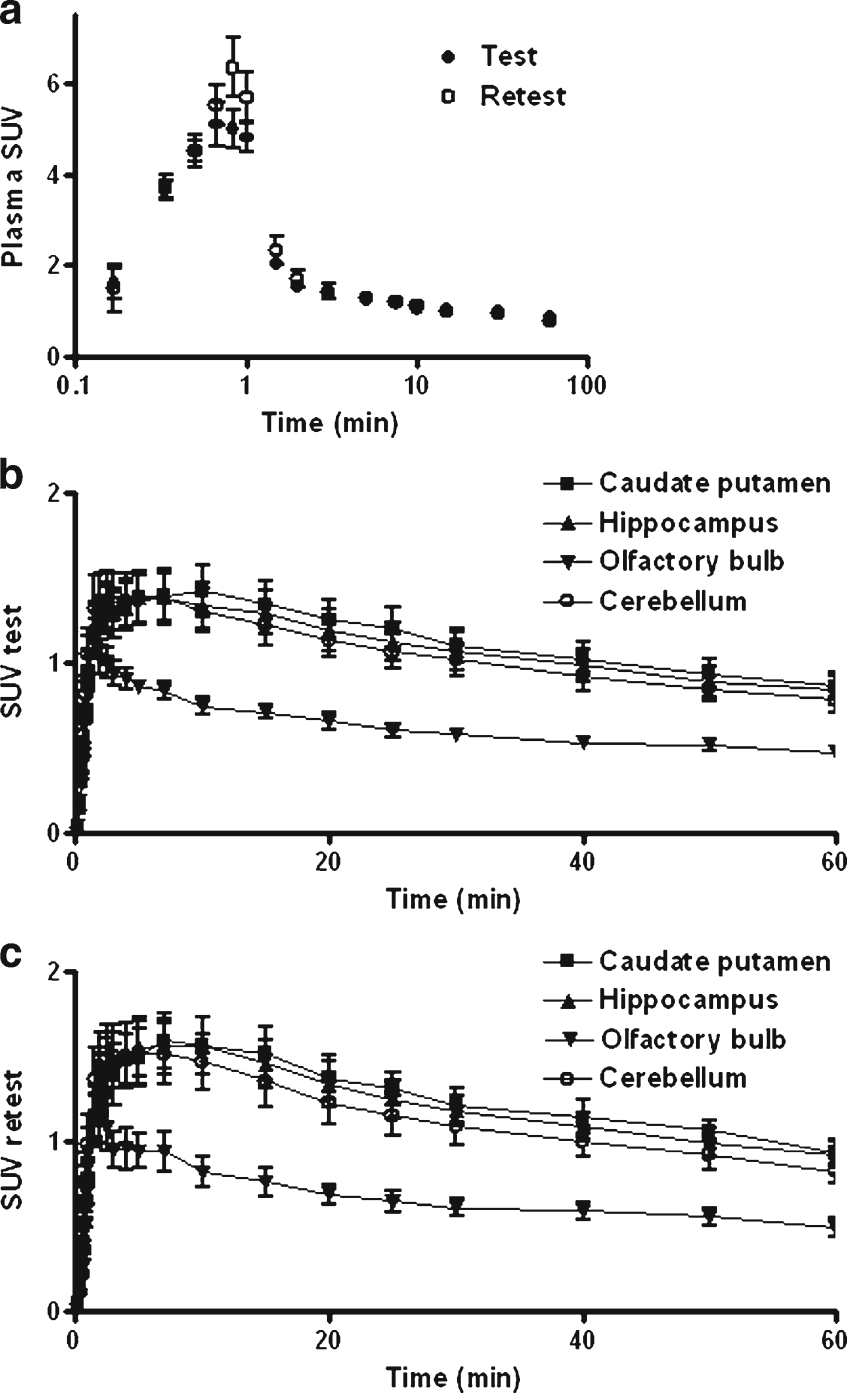
**a** Average plasma input curve for the test and retest scans. Time-activity curves for different brain regions in the **b** test and **c** retest scans. *Error bars* indicate SD.

The catheter in a superficial branch of the femoral artery allowed rapid blood sampling: 0.1 ml of blood could be drawn within 10 s. Time-activity curves of radioactivity in arterial plasma during the test and retest scans are shown in Fig. 3a. For the sake of clarity, the data are plotted on a logarithmic *X*-axis. Both curves show a peak about 1 min after the start of the infusion pump followed by a slow wash-out as observed previously [18]. Plasma radioactivity at 0.83 and 1 min appears to be slightly greater in scan 2 than in scan 1, but all other data points overlap and the two curves are not statistically different. Sampling from the main femoral artery or sampling from the superficial branch of this artery during the retest scan produced identical results, as expected.

### PET Images

**Fig. 4:**
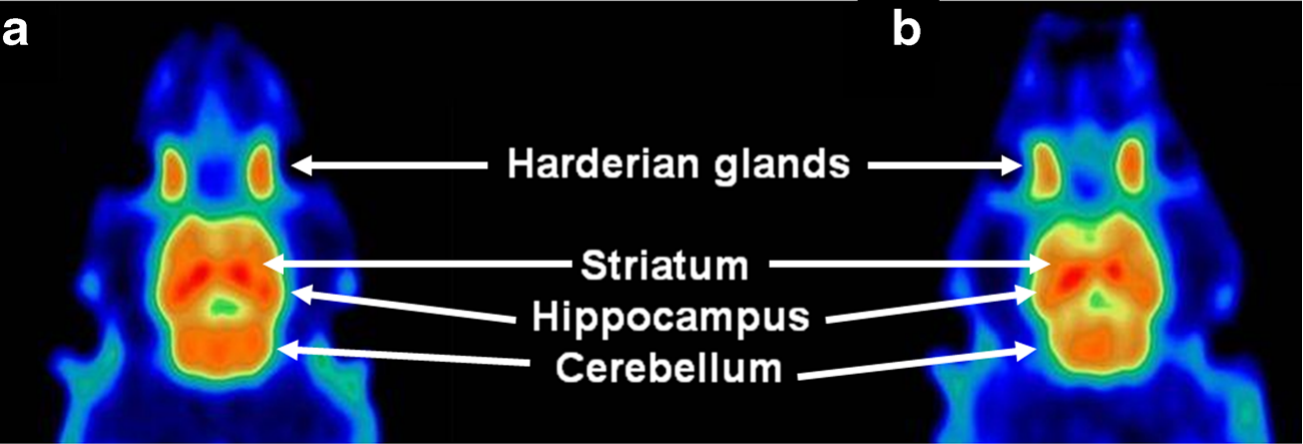
MicroPET images of a single rat acquired during the **a** test and **b** retest scans. A dose of 59.9 and 41.8 MBq of [^11^C]MPDX was injected on these two occasions. *Images* represent summed data of all dynamic frames. The position of several brain regions and of the Harderian glands is indicated by *arrows*.

Representative images of adenosine A1 binding in the rat brain during the test and retest scan are presented in Fig. 4. The images show high tracer uptake in the hippocampus, striatum, and cerebellum. Lower levels of radioactivity are observed in other brain areas such as cerebral cortex and the olfactory bulb. Similar images were acquired in previous studies from our institution [7, 18, 19]. Visual differences between scan 1 and scan 2 were not detected. Time-activity curves for different brain regions show slightly higher results in the retest scan but the differences are not significant (Fig. 3b, c). Biodistribution SUV values (acquired after the retest scan) are presented in the supplementary data. These were comparable with previously reported values for [^11^C]MPDX in our institution [18, 19].

### Test-Retest Reproducibility

Injected tracer dose, specific radioactivity, and tracer purity in the test and retest scans were not significantly different. Data for regional *V*_T_ (calculated by Logan graphical analysis, using PET images fused with a MRI template) are shown in Table 1. Statistically significant differences between *V*_T_ values acquired during test and retest were not observed. The relative difference between scan 1 and scan 2 was <5 % in all brain regions, with exception of the olfactory cortex, cortex, hypothalamus, and olfactory bulb where values between 5 and 10 % were observed. The TRV is for all brain regions <10 % with exception of the olfactory cortex. The COV for *V*_T_ showed an average for all brain regions of 10.9 ± 2.2 % in scan 1 and 12.5 ± 3.1 % in scan 2. The reliability of the measurements between and within subjects, expressed as ICC, indicated a substantial agreement between test and retest, with average value of 0.86 ± 0.11, and almost a perfect agreement in most regions, with ICC values greater than 0.9 [17].

**Table 1:** Reliability of estimates of regional tracer distribution volume (*V*_T_)

Region	Test	Retest	% relative difference	TRV	COV (%) test	COV (%) retest	ICC
Whole brain	1.11 ± 0.13	1.14 ± 0.14	2.28 ± 4.69	3.7 ± 3.2	11.5	12.0	0.96
Amygdala	0.90 ± 0.08	0.94 ± 0.09	4.58 ± 7.61	6.1 ± 5.4	8.4	9.2	0.79
Caudate putamen	1.29 ± 0.17	1.35 ± 0.22	3.76 ± 6.25	4.7 ± 4.9	13.1	16.2	0.95
Mesencephalic region	1.09 ± 0.10	1.13 ± 0.15	3.40 ± 8.63	6.9 ± 5.2	9.5	13.5	0.83
Pons	0.97 ± 0.07	1.00 ± 0.10	3.59 ± 8.46	7.1 ± 4.9	7.5	9.6	0.69
Medulla	0.90 ± 0.08	0.93 ± 0.09	3.21 ± 7.57	6.5 ± 3.9	8.5	9.4	0.79
Cerebellum	1.17 ± 0.14	1.18 ± 0.12	1.33 ± 4.12	2.8 ± 2.9	12.4	10.1	0.98
Olfactory bulb	0.66 ± 0.10	0.70 ± 0.14	7.06 ± 13.15	9.2 ± 9.3	14.5	19.6	0.84
Cortex	1.09 ± 0.14	1.10 ± 0.14	1.41 ± 4.69	3.0 ± 3.4	13.1	12.9	0.98
Hippocampus	1.26 ± 0.12	1.28 ± 0.13	2.13 ± 4.77	3.8 ± 3.1	9.8	9.9	0.94
Hypothalamus	0.94 ± 0.07	1.01 ± 0.12	7.82 ± 8.60	9.2 ± 5.3	8.0	11.5	0.69
Thalamus	1.41 ± 0.18	1.42 ± 0.17	0.60 ± 4.29	2.9 ± 2.8	12.8	11.9	0.98
Olfactory cortex	0.97 ± 0.11	1.06 ± 0.17	9.09 ± 12.37	10.3 ± 8.8	11.5	16.3	0.74
Brain remnants	1.21 ± 0.14	1.24 ± 0.16	2.88 ± 6.55	4.9 ± 4.4	11.8	12.6	0.93
Mean ± SD			3.80 ± 2.54	5.8 ± 2.5	10.9 ± 2.2	12.5 ± 3.1	0.86 ± 0.11

PET images were co-registered with a MRI template in order to identify regions-of-interest

*TRV* test-retest variability, *COV* coefficient of variation, *ICC* intraclass correlation coefficient

We also examined the reproducibility of *V*_T_ data calculated after co-registration of the PET data with a [^11^C]MPDX PET template (Table 2). The reliability of *V*_T_ estimations using this approach was quite similar to that of estimations using a MRI template.

**Table 2:** Reliability of estimates of regional tracer distribution volume (*V*_T_)

Region	Test	Retest	% relative difference	TRV	COV (%) test	COV (%) retest	ICC
Whole brain	1.11 ± 0.12	1.15 ± 0.14	2.99 ± 5.43	4.2 ± 3.9	11.0	12.0	0.94
Amygdala	0.84 ± 0.08	0.90 ± 0.10	6.60 ± 8.57	8.6 ± 4.9	9.0	11.3	0.74
Caudate putamen	1.29 ± 0.17	1.32 ± 0.20	2.43 ± 4.85	3.6 ± 3.6	13.1	15.4	0.97
Mesencephalic region	1.07 ± 0.10	1.10 ± 0.16	2.58 ± 7.88	6.0 ± 4.8	9.7	14.5	0.88
Pons	0.93 ± 0.07	0.97 ± 0.11	4.49 ± 8.29	7.3 ± 4.7	7.6	10.8	0.73
Medulla	0.88 ± 0.07	0.92 ± 0.10	5.24 ± 9.39	8.5 ± 5.0	8.3	10.8	0.65
Cerebellum	1.17 ± 0.11	1.22 ± 0.11	4.25 ± 6.28	4.6 ± 5.3	9.7	8.8	0.87
Olfactory bulb	0.66 ± 0.10	0.69 ± 0.13	4.00 ± 7.79	6.0 ± 5.3	15.1	19.3	0.93
Cortex	1.11 ± 0.14	1.13 ± 0.14	1.86 ± 4.59	3.2 ± 3.3	12.9	12.8	0.97
Hippocampus	1.23 ± 0.11	1.28 ± 0.14	3.85 ± 6.88	6.2 ± 3.9	9.1	10.7	0.86
Hypothalamus	0.89 ± 0.08	0.95 ± 0.13	6.76 ± 9.67	9.4 ± 5.1	8.6	13.2	0.71
Thalamus	1.39 ± 0.17	1.43 ± 0.18	2.67 ± 5.27	4.7 ± 2.9	12.0	12.5	0.95
Olfactory cortex	0.94 ± 0.11	1.02 ± 0.17	7.48 ± 10.44	8.6 ± 7.7	12.0	16.8	0.83
Brain remnants	1.20 ± 0.14	1.23 ± 0.15	2.81 ± 6.15	4.4 ± 4.3	11.7	12.1	0.94
Mean ± SD			4.14 ± 1.78	6.1 ± 2.1	10.7 ± 2.2	12.9 ± 2.8	0.85 ± 0.11

PET images were co-registered with an [^11^C]MPDX PET template in order to identify regions-of-interest

*TRV* test-retest variability, *COV* coefficient of variation, *ICC* intraclass correlation coefficient

### Factors Affecting the Reliability of *V*_T_ Estimates

**Fig. 5:**
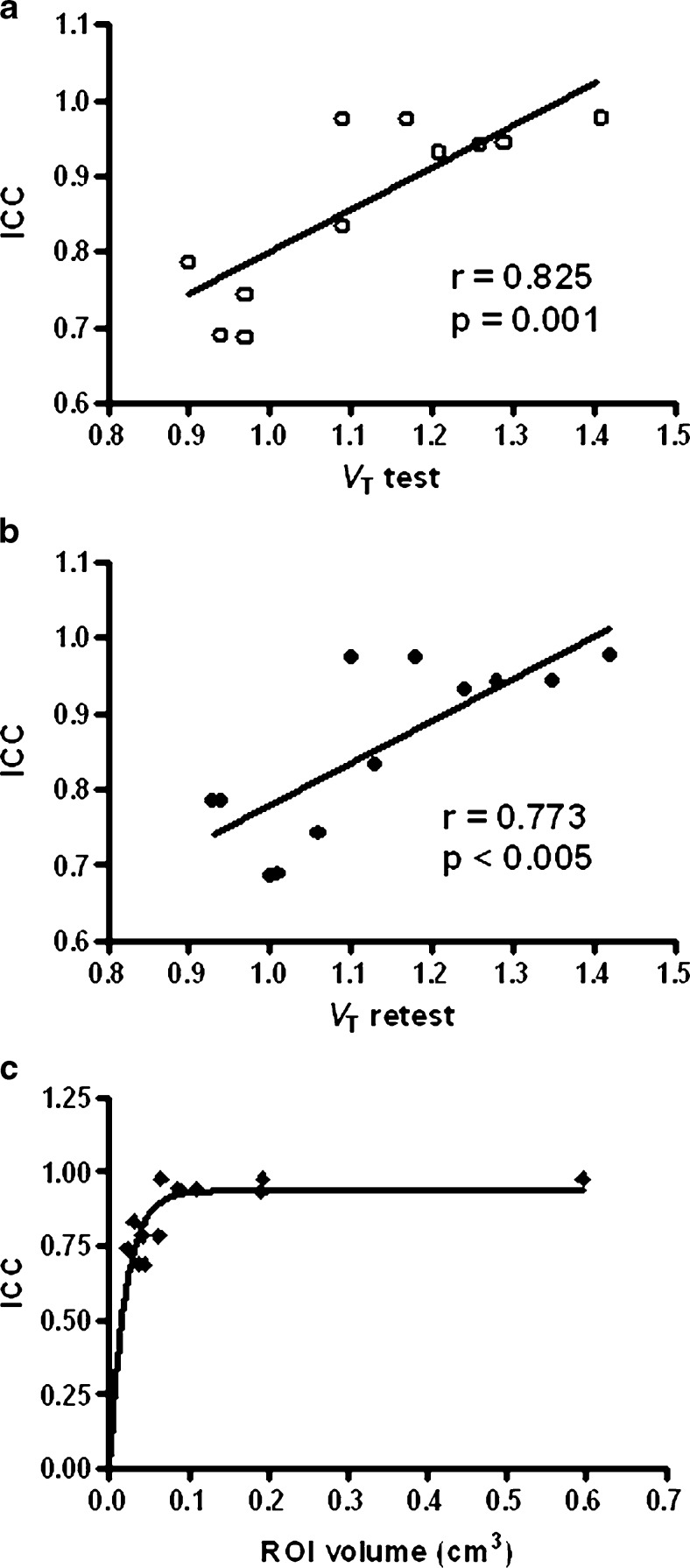
Correlation between tracer distribution volume in a region-of-interest and the value of the ICC for **a** test and **b** retest and the relationship between ROI volume (in cm^3^) and the value of **c** ICC.

ICC data showed a significant positive correlation with tracer distribution volume (*V*_T_-value) in the region-of-interest (ROI), both in the test (*r* = 0.83, *p* = 0.001) and retest (*r* = 0.77, *p* < 0.005) scans (Fig. 5a, b). ROI volume (in cm^3^) appeared to affect *V*_T_ reliability as well. A ROI volume of 0.02 cm^3^ is at least needed to get a moderate agreement. ICC is not affected with a ROI volume of 0.10 cm^3^ or higher (Fig. 5c).

### Model Fits *vs* Graphical Analysis

**Fig. 6:**
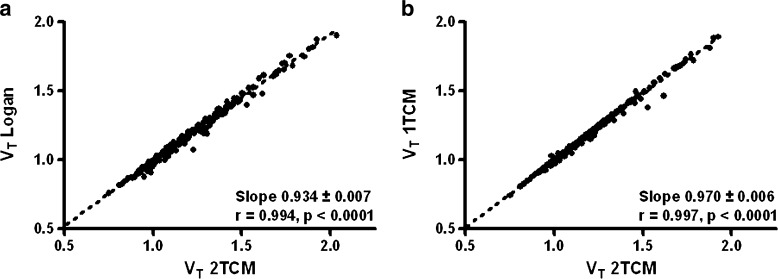
Correlation between **a** *V*_T_ Logan and *V*_T_-2TCM and **b** *V*_T_-1TCM and *V*_T_-2TCM.

*V*_T_ data obtained from Logan graphical analysis, a 1TCM fit, or a 2TCM fit show a striking resemblance which is demonstrated in Fig. 6. With slopes of 0.93 and 0.97 and correlation coefficients of 0.99 and 1.00, the data were almost identical (Fig. 6).

A recent publication suggested that the olfactory bulb may be used as a reference region to estimate A1 adenosine receptor (A_1_AR) availability in the rodent brain without any need for arterial blood sampling [12]. Since our own data indicated that olfactory bulb was indeed the brain region showing the lowest uptake of [^11^C]MPDX, we tried a SRTM fit for estimation of *BP*_ND_ from our [^11^C]MPDX data. Although the relative differences between *BP*_ND_ estimated in the test and retest scans were comparable to those observed for *V*_T_, SD values were in all regions much higher. TRV (17.2 ± 8.2) and COV for the test (23.6 ± 10.1) and retest (17.7 ± 10.5) scan were higher compared to the values observed for *V*_T_ calculated by Logan graphical analysis. The average ICC (0.41 ± 0.26) for *BP*_ND_ indicated a fair agreement, with only one brain region showing almost perfect agreement [17]. An overview of quality parameters for the SRTM fit is presented in the supplementary data [20].

## Discussion

Rodents can be catheterized in different ways, for example, by placement of cannulas in the aorta, a femoral artery, or a carotid artery. Catheterization of a femoral artery is frequently applied [4–6]. Catheterization of a superficial branch of a femoral artery offers the same benefits but makes repeated arterial blood sampling in a single rat possible. In order to determine if such catheterization causes significant adverse effects, we examined different aspects of animal welfare such as weight loss, bleeding, visible infections, and disturbances of movement. These observations indicated that the surgery had negligible adverse effects. However, we did not examine physiological or metabolic parameters of inflammation which could be altered by our technique. Such parameters should be explored in the future studies.

Catheterization of a superficial branch of a femoral artery could be applied in longitudinal PET studies involving maximally three quantitative scans with rapid blood sampling. A catheter could be placed in a superficial artery in the left and right hind limbs (for the first and second scan) and in one of the large femoral arteries (for the last scan), after which the rat would reach its humane end point.

A recent publication describes continuous measurements of blood pressure and heart rate, using a permanent catheter inserted in a femoral artery [21]. This approach could be used if more than three PET scans with blood sampling are required, but the impact of a permanent catheter on neuroreceptor availability in the brain has to be explored.

Catheterization of a superficial branch of the femoral artery requires the use of a catheter with a smaller diameter than is normally used for arterial sampling (0.28 mm as compared to 0.40 mm inner diameter). For this reason, we initially questioned whether rapid blood sampling would be possible with this technique. However, we noticed that blood samples of normal size (0.1–0.15 ml) could be drawn within a period of 10 s. Apparently, the pressure in the artery is sufficient for rapid sampling through a catheter with a narrow bore.

Tracer uptake, *i.e*., the density of adenosine A_1_AR in a brain region of interest, appears to be an important factor determining the reliability of *V*_T_ estimates. Figure 5 indicates a significant positive correlation between the intraclass correlation coefficient and *V*_T_ value in the range 0.9 to 1.5. *V*_T_ reproducibility appears to be affected also by ROI volume if volumes are smaller than 0.10 cm^3^. Figure 5 indicates an excellent reproducibility for volumes greater than this threshold, but at very small volumes, the reliability of the measurements is impaired. The observed volume threshold may be tracer- and camera-dependent. More research in this area is needed.

The data reported in Fig. 6 indicate that *V*_T_ can be reliably estimated using either a 1TCM fit, a 2TCM fit, or Logan graphical analysis. However, the use of graphical analysis results in a slight (7 %) underestimation of *V*_T_ as compared with the use of a 2TCM fit. This difference is probably related to well-known limitations of the Logan plot [22, 23].

We were not able to estimate *BP*_ND_ from a 2TCM fit, since erratic and abnormal values were acquired in most of the cases (an overview of quality parameters for the 2TCM fit is presented in the supplementary data). This may be due to the study design and the resulting shape of the cerebral TACs. In a previous study where [^11^C]MPDX was injected manually as a rapid bolus [7], it proved possible to estimate *BP*_ND_ from a 2TCM fit. In the present study, we injected the tracer as a slow bolus (1 ml/min), using an infusion pump. This slow bolus improved the reproducibility of the plasma time-activity curves but altered the kinetics of the tracer within the brain. Because of these altered kinetics, the 2TCM fit may have provided a model solution which was neither optimal nor unique.

The data reported in Table 3 indicate a poor reproducibility of *BP*_ND_ values for [^11^C]MPDX calculated from a SRTM fit with olfactory bulb as the reference region. The use of olfactory bulb as a reference appears to also result in an underestimation of whole brain *BP*_ND_ of more than 50 % when the current values are compared with previously published arterial-input pharmacokinetic modeling data [7, 12]. Both this poor reproducibility and the underestimation may be due to the fact that the olfactory bulb is not a good reference region for [^11^C]MPDX. In blocking experiments which involved the specific A_1_AR antagonist DPCPX, [^11^C]MPDX uptake (SUV) in the olfactory bulb was significantly reduced from 0.64 ± 0.18 to 0.34 ± 0.07 [7]. Thus, about 47 % of the bulbar [^11^C]MPDX uptake appears to represent specific binding to A_1_AR.

**Table 3:** Reliability of *BP*_ND_ calculated from a simplified reference tissue model (SRTM) fit, with the olfactory bulb as reference region

Region	Test	Retest	% relative difference	TRV	COV (%) test	COV (%) retest	ICC
Whole brain	0.70 ± 0.11	0.66 ± 0.07	−2.79 ± 16.20	10.8 ± 14.2	15.8	10.7	0.34
Amygdala	0.39 ± 0.38	0.18 ± 0.13	9.02 ± 40.92	31.4 ± 24.1	45.6	33.7	0.44
Caudate putamen	0.96 ± 0.93	0.17 ± 0.06	−1.68 ± 11.35	8.1 ± 8.6	17.6	7.0	0.69
Mesencephalic region	0.68 ± 0.64	0.12 ± 0.09	−3.68 ± 18.41	15.1 ± 12.1	17.3	13.5	0.13
Pons	0.51 ± 0.48	0.12 ± 0.09	−1.78 ± 26.39	22.5 ± 13.0	23.5	18.0	0.02
Medulla	0.40 ± 0.38	0.14 ± 0.08	5.81 ± 48.42	34.1 ± 28.9	35.6	21.2	*
Cerebellum	0.77 ± 0.73	0.10 ± 0.14	−3.72 ± 20.25	16.5 ± 14.5	12.8	19.2	0.34
Cortex	0.65 ± 0.61	0.11 ± 0.07	−4.39 ± 16.38	13.0 ± 12.9	16.3	11.4	0.24
Hippocampus	0.93 ± 0.90	0.18 ± 0.09	−1.46 ± 18.79	13.5 ± 14.8	19.5	10.3	0.24
Hypothalamus	0.45 ± 0.49	0.17 ± 0.19	13.17 ± 31.95	22.4 ± 16.7	37.0	38.8	0.73
Thalamus	1.14 ± 1.08	0.25 ± 0.09	−2.67 ± 19.54	14.4 ± 16.3	21.8	8.5	0.33
Olfactory cortex	0.48 ± 0.52	0.14 ± 0.16	10.10 ± 15.42	13.4 ± 8.7	28.5	29.8	0.93
Brain remnants	0.84 ± 0.81	0.12 ± 0.06	−1.82 ± 12.07	8.1 ± 9.6	14.9	7.8	0.51
Mean ± SD			1.09 ± 6.11	17.2 ± 8.2	23.6 ± 10.1	17.7 ± 10.5	0.41 ± 0.26

PET images were co-registered with a MRI template in order to identify regions-of-interest

*TRV* test-retest variability, *COV* coefficient of variation, *ICC* intraclass correlation coefficient

*ICC calculation gave an incorrect solution. *N* = 5

## Conclusion

Repeated rapid arterial blood sampling is possible with our new surgical procedure. This allows longitudinal studies in rats involving repeated quantitative PET imaging with a high test-retest reproducibility.
